# Impact factor: Playing a number on you

**DOI:** 10.1017/qpb.2025.10024

**Published:** 2025-11-14

**Authors:** Olivier Hamant

**Affiliations:** Laboratoire de Reproduction et Développement des Plantes, Université de Lyon, ENS de Lyon, UCBL, INRAE, CNRS, 46 Allée d’Italie, Lyon, Cedex 07, France

**Keywords:** Goodhart law, impact factor, publishing, quantitative plant biology, systems thinking

## Abstract

The impact factor has become a defining feature of scientific journals. However, such reductionism can be toxic to science. As Cambridge University Press *Quantitative Plant Biology* celebrates its 5-year anniversary, and its first impact factor, this is an opportunity to set things straight. A call to value what a scientific journal is about: a community of scientists, a guarantee of rigour and quality, an invitation to explore the complexity of our world, a fair and ethical environment and an engaging, diverse and creative arena.

## Graphical abstract

**Figure:**
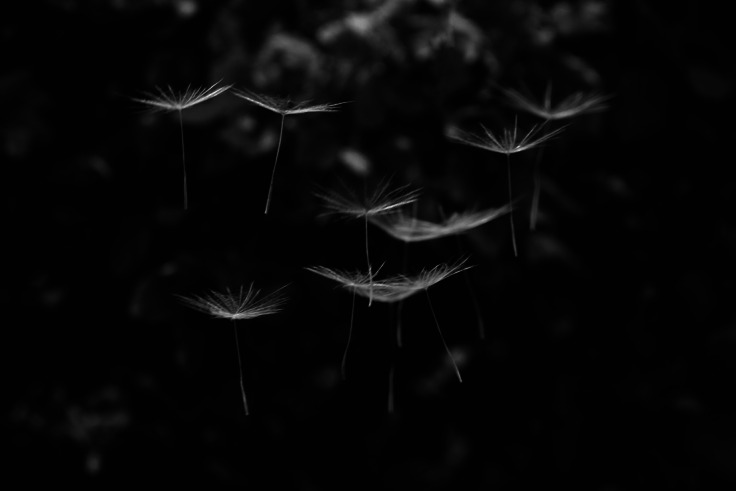

“Impact” – This word is not without connotations. For instance, if I wanted to get a stronger impact with a newspaper, I would choose the thickest and largest format, I would roll it up and I would certainly be able to generate an impact as I hammer it on a fly. Impact is first and foremost a violent term.

Of course, today, when a journal gets an ‘impact factor’, that military rhetoric is forgotten, probably because many of us also forgot what war is about (Figure 1). The impact factor is a ranking tool for scientific journals. It was created by Eugene Garfield at the Institute for Scientific Information (ISI) in Philadelphia, USA. The first impact factors appeared in 1975, concomitantly with the rise of the economic neoliberal model, and consistently with the obsession of scientists for metrics. In short, the impact factor is ‘a measure of the frequency with which the “average article” in a journal has been cited in a particular year or period (…) The [two-year] impact factor of a journal is calculated by dividing the number of current year citations to the source items published in that journal during the previous two years’ (ISI, 1994).

**Figure 1.**
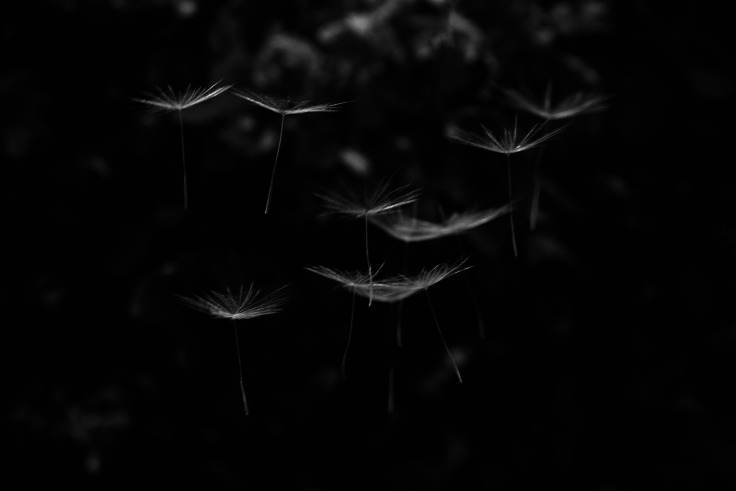
Dandelion’s crested achenes are flying and waiting for impact. Credit photo: Hasan Almasi (Unsplash, 3 May 2018, Canon, EOS 7D).

Because this tool provides a number, it also fuels reductionist thinking: one may assign the complex value of a journal to a single number. This can be pernicious. In fact, this is a well-established fact, whatever the metrics. The Goodhart law says it all: ‘when a measure becomes a target, it ceases to be reliable’. The race for a high impact factor is no exception. It has produced:

- an inflation in the number of scientific publications (since more publications mean more citations),
- an increased pace of publications (to be able to produce more publications),
- and as a result, an increased conservatism in scientific discovery in the past decades, as shown by Park et al., in a seminal article (Park et al., 2023).

This race for producing and publishing articles in high-impact-factor journals has also facilitated many problematic feedback loops:

- an increased rate of fraud in scientific publications (because the increased pace reduces the ability of reviewers to control the contents) (Fang et al., 2012; Richardson et al., 2025),
- the proliferation of predatory journals, where (financial) impact prevails over (scientific) rigour and ethics (Laine et al., 2025),
- the definition of salaries and bonuses based on publications, using the impact factor as a rewarding tool (Owen, 2019),
- the scientific publishing financial boom (e.g., when Elsevier’s profits outperformed those of Apple, Google or Amazon in 2010) (Buranyi, 2017), with important biases in what gets to be published and where.

As Cambridge University Press *Quantitative Plant Biology* reaches its 5th anniversary in 2025, it also got its first impact factor. As one can imagine from this preamble, we are welcoming this news with mixed feelings.

On the one hand, this is a recognition of the journal belonging to a larger scientific community and a measure of the citations across the scientific literature. *Quantitative Plant Biology*’s impact factor reached 2.5 in 2025, and we are thrilled to see such dynamics.

On the other hand, that number ignores what is important to us – that is, what one can find in the journal:

- New angles on noise (Bhalerao, 2024), on somatic mutations in trees (Iwasa et al., 2024), on intermittent stresses (Gama et al., 2025) or on light heterogeneity (Claydon et al., 2025);
- New resources to study plant morphogenesis – for example, for root posture (Yagi et al., 2024), for zygote growth (Kang et al., 2024), for leaf growth with a quantitative atlas (Tolleter et al., 2024), for sepal curvature with a morphospace (Battu et al., 2025), for stem shape with four-dimensional phenotyping (Yoshida et al., 2025), for gynoecium growth with time-lapse imaging (Wodniok, 2025) and with many deep-learning methods (Hong et al., 2025; Wanner et al., 2024);
- The strong emphasis on systems thinking – for example, regarding patterning and micromechanics (Jacobs et al., 2025), synthetic gene circuits (Khan & Lister, 2025), fruit growth (Chung et al., 2025) or hypoxia (Chen et al., 2025);
- A collection of review articles on ion homeostasis in plant cells (Dreyer et al., 2024; Contador-Álvarez et al., 2025), with independent focuses on silicon (Huang & Ma, 2024), phosphorus (Chiang et al., 2025), calcium (Brownlee & Wheeler, 2025), zinc (Krämer, 2025), potassium (Wegner et al., 2025), iron (Leskova et al., 2025), manganese (Meier et al., 2025) or chloride (Silva-Herrera et al., 2025);
- Post-normal scientific themes, such as tools to avoid human biases when analysing patterns (Galipot, 2025) and a call for participatory approaches (Correa et al., 2025);
- Societal applications from quantitative plant biology – for example, for the maple syrup industry (McNulty et al., 2025);
- Last but not least, the authors – many world experts in their fields – and their creativity, with tributes to seminal past articles (e.g., on Turing (Siero & Deinum, 2025)).

In addition to content, the impact factor also overlooks the essentials of the journal:

- a community-based editorial board,
- a not-for-profit publisher (Cambridge University Press) and partner institution (John Innes Centre),
- a rigorous reviewing process,
- strong ethics (fully open access, free waivers available and community).

In the end, rather than using data to provide a number (reductionist path), our journal is all about quantitative thinking – that is, just the opposite: how to use numbers in the data to provide new questions (systemic path). Thus, our goal is not to reduce biology to numbers, but rather to use numbers to expand our understanding of the complexity and richness of the plant world. As we celebrate the journal’s anniversary, we would prefer to move away from impacting the reader with a factor, but instead call for a pact with our readers to broaden the exploration of the plant world and its many surprises.

## Data Availability

No data and code are available for this manuscript.
