# Supplementary material for: Impact factor: Playing a number on you
Source: Quant Plant Biol. 2025 Nov 14;6:e33. doi: 10.1017/qpb.2025.10024 (PMC12658294; doi:10.1017/qpb.2025.10024)
Supplement: Peer review history

# Editor report: Recommendation: Impact factor: Playing a number on you — R0/PR2

**DOI:** 10.1017/qpb.2025.10024.pr2

**Affiliations:** John Innes Centre, United Kingdom of Great Britain and Northern Ireland

Dear Olivier, 

Thank you for this timely and thoughtful editorial. 

With best wishes

Richard

# Editor report: Decision: Impact factor: Playing a number on you — R0/PR3

**DOI:** 10.1017/qpb.2025.10024.pr3

**Affiliations:** John Innes Centre, United Kingdom of Great Britain and Northern Ireland

No accompanying comment.
